# EEG Feature Extraction and Classification for Upper Limb Flexion and Extension Motor Imagery Based on Discriminative Filter Bank Common Spatial Pattern

**DOI:** 10.3390/brainsci16020217

**Published:** 2026-02-11

**Authors:** Yuqi Zhang, Xiaoyan Shen

**Affiliations:** School of Information Science and Technology, Nantong University, Nantong 226019, China; 2330310036@stmail.ntu.edu.cn

**Keywords:** electroencephalogram (EEG), upper limb motor imagery (MI), common spatial pattern (CSP), multilayer perceptron (MLP)

## Abstract

Background: Traditional common spatial pattern (CSP) algorithms for upper limb neural rehabilitation face inherent challenges of overlapping cortical representations and frequency sensitivity, which hinder the decoding performance of motor imagery (MI) electroencephalogram (EEG) signals. Objective: To address these issues, this study adopts an improved discriminative filter bank CSP (DFBCSP) framework and applies it to the decoding of upper limb MI-EEG signals, achieving remarkable classification performance. Methods: EEG data were acquired from sixteen participants performing two-class (left upper limb flexion-extension vs. relaxing) and three-class (left upper limb flexion vs. right upper limb extension vs. relaxing) MI tasks. The acquired EEG data were then decomposed into nine distinct sub-bands, followed by the adoption of a mutual information-based feature selection strategy to optimize the feature sets. These optimized feature sets were subsequently input into three classification models, namely multilayer perceptron (MLP), support vector machine (SVM), and linear discriminant analysis (LDA), for MI task classification. Results: Experimental results demonstrate that the DFBCSP + MLP method significantly outperforms the traditional CSP approach. Specifically, it achieves an accuracy of 94.83% (Kappa coefficient: 0.890) in two-class MI tasks and 86.20% (Kappa coefficient: 0.775) in three-class MI tasks. Conclusion: The DFBCSP + MLP framework exhibits high robustness and provides a potential technical framework and theoretical basis for future research on the rehabilitation of patients with upper limb motor dysfunction.

## 1. Introduction

In recent years, the incidence of limb disability and paralysis resulting from amyotrophic lateral sclerosis (ALS) and spinal cord injury (SCI) has been steadily increasing. Patients with motor disabilities have intact cognitive functions but lack voluntary control over their muscles and the peripheral nervous system, making it impossible for them to effectively control their body trunk through brain signals. Although traditional clinical approaches have achieved certain progress in limb function recovery, they still struggle to fully restore complex motor functions. Brain–Computer Interface (BCI) technology offers the possibility of establishing a bidirectional communication pathway between the brain and external devices. By acquiring and decoding neurophysiological signals such as Electroencephalography (EEG), this technology can directly convert the subject’s motor intentions into physical commands, thereby enabling the control of assistive devices [1,2]. This technology brings new hope to patients with motor impairments but intact cognitive abilities.

In neural rehabilitation, the Motor Imagery (MI) experimental paradigm is widely used as a control source due to its ability to map active intentions without requiring actual limb movements. It not only promotes the repair or reconstruction of damaged motor pathways and awakens some dormant neural synapses but also can interact with actual movements to achieve better motor cortex reorganization effects [3]. However, distinguishing between different fine movements within the upper limb (Extension and Flexion) still poses significant challenges [4]. The representational areas of such movements in the cerebral cortex highly overlap, and EEG signals are characterized by low signal-to-noise ratio (SNR) and significant individual differences, which place extremely high demands on the discriminative power of feature extraction algorithms.

Commonly utilized feature extraction methods for MI can be categorized into several primary types based on the nature of the classification features. First, time–domain analysis focuses on waveform characteristics as they evolve over time, employing techniques such as statistical features (mean, variance, and standard deviation) [5], autoregressive (AR) models [6], and event-related potentials (ERP) [7]. Second, frequency–domain analysis investigates EEG signal characteristics across different frequency bands corresponding to various physiological states, utilizing methods such as power spectral density (PSD) [8,9], differential entropy (DE) [10], and energy ratios [11]. Thirdly, spatial–domain analysis focuses on exploring the activation relationships among different brain regions, mainly extracting features through methods such as Common Spatial Pattern (CSP) [12,13], Independent Component Analysis (ICA) [14], and Surface Laplacian (SL) [15]. In addition, time-frequency analysis can be performed to capture the time-frequency domain features of EEG signals using techniques including Wavelet Transform (WT) [16] and Hilbert-Huang Transform-Empirical Mode Decomposition (HHT-EMD) [17]. MI-EEG signals exhibit the most distinct spatial distribution differences, making the CSP widely used for extracting discriminative spatial features. Numerous researchers have conducted extensive studies to derive more prominent CSP features [18]. For instance, Ana [19] proposed an adaptive CSP (ACSP) algorithm, which significantly reduced the training time required for new subjects and achieved successful three-class classification for complex MI tasks. Tang et al. [20] proposed a method based on the Bhattacharyya distance to select the optimal frequency band for each electrode across different subjects, followed by feature extraction using an improved B-CSP algorithm to achieve the classification of motor imagery tasks. Fu et al. [21] addressed the issue that the CSP algorithm repeatedly selects feature patterns in the feature space by proposing a sparse CSP algorithm. This approach embeds sparse technology and iterative search into the CSP framework, selecting EEG signals from a few channels with the most prominent features and thereby improving the accuracy of feature classification. Peterson V et al. [22] proposed a novel classification method integrating multi-band and time window techniques. This method extracts features from each frequency band using the CSP algorithm and incorporates a priori discriminative information into the model via a fast feature selection and classification approach based on elastic net regression, thereby improving the classification accuracy of MI-based BCI systems. Convolutional networks exhibit excellent automatic feature extraction capability in complex signal processing scenarios [23]. Fu et al. [24] proposed a convolutional transformer network integrated with an adaptive learning module, which not only enhances the individual motor imagery classification performance but also shortens the calibration time for new subjects.

The CSP is a spatial filtering feature extraction algorithm designed for binary classification tasks. It is capable of extracting the spatial distribution components of each class from multi-channel BCI data. But the traditional CSP algorithm is highly sensitive to noise, and its performance is significantly dependent on the selection of frequency bands [25]. To address these limitations, we introduce the discriminative filter bank common spatial pattern (DFBCSP), which enables the precise elimination of redundant information while retaining features with the highest discriminative contribution. We aim to explore the potential of the DFBCSP algorithm in the classification of EEG signals during upper-limb extension and flexion MI. We developed a feature extraction model based on DFBCSP and integrated it with classification techniques, including multi-layer perceptron (MLP), support vector machine (SVM), and linear discriminant analysis (LDA), to analyze EEG signals from subjects performing upper-limb imagery tasks. By comparing with the traditional CSP, this study evaluates the classification performance of the DFBCSP in upper limb motor imagery tasks using statistical metrics such as classification accuracy, Kappa value, and Receiver Operating Characteristic (ROC) curve.

## 2. Materials and Methods

**Figure 1:**
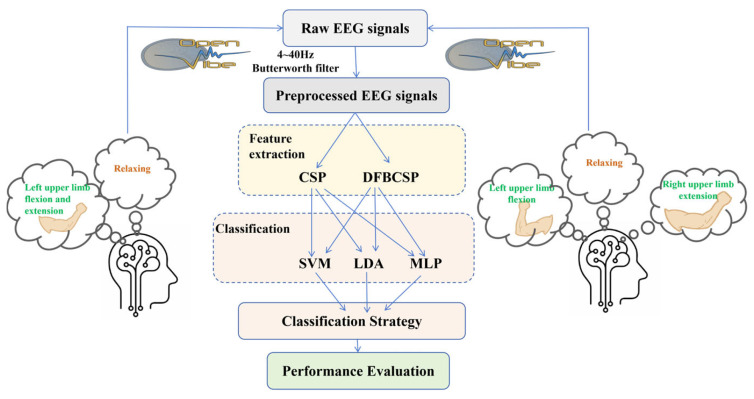
Basic steps of research content.

The study encompasses three core components: EEG signal acquisition, signal processing, and classification performance evaluation. The detailed implementation procedures are illustrated in Figure 1, and the entire workflow was executed using the OpenViBE platform (https://openvibe.inria.fr, OpenViBE 3.5.0).

### 2.1. Subjects, Data Acquisition, and Experimental Procedure

Sixteen healthy participants (labeled S1–S16) were recruited for the study, comprising ten males and six females with an age range of 21–27 years. All subjects were right-handed and possessed normal or corrected-to-normal vision. Furthermore, none of the participants reported a history of psychiatric or neurological disorders.

**Figure 2:**
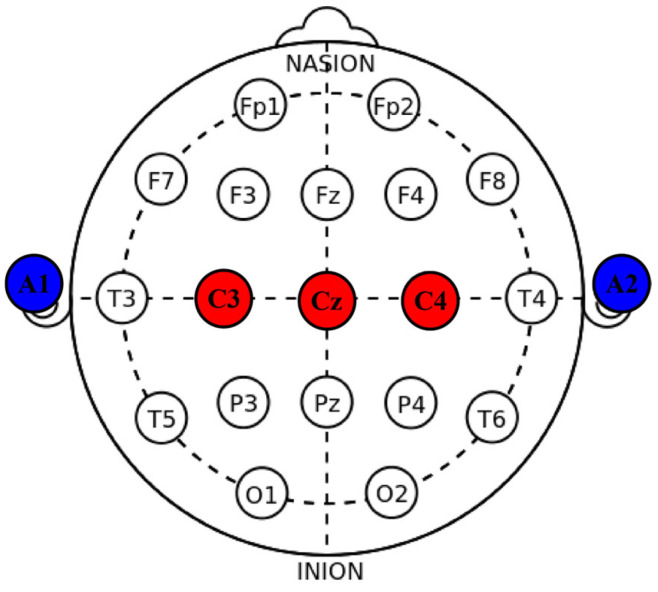
Electrode distribution.

The OpenBCI platform (openbci.com, New York) was selected for EEG signal acquisition, with the sampling rate configured at 250 Hz. The electrode arrangement strictly adhered to the International 10–20 system, as illustrated in Figure 2. The red regions represent the data acquisition electrodes, and the blue regions denote the reference electrodes positioned at the earlobes. When subjects perform unilateral limb Motor Imagery (MI) tasks, regular potential changes are generated in relevant regions of the cerebral cortex. Specifically, the *μ* rhythm (8–12 Hz) and *β* rhythm (13–30 Hz) in the contralateral primary sensorimotor cortex exhibit a significant decrease in energy, known as Event-Related Desynchronization (ERD); simultaneously, the energy of the corresponding rhythms in the ipsilateral regions increases significantly, referred to as Event-Related Synchronization (ERS) [26]. Therefore, only C3, Cz, and C4, which are most closely associated with the primary sensorimotor cortex, were selected as measurement channels in this experiment. This streamlined channel scheme not only effectively reduces computational load but also significantly improves the subject’s wearing comfort. Prior to data collection, to optimize signal transmission quality, experimenters need to clean the subject’s scalp with medical alcohol and apply conductive gel to minimize electrode impedance, thereby ensuring that the collected EEG signals have a high SNR.

**Figure 3:**
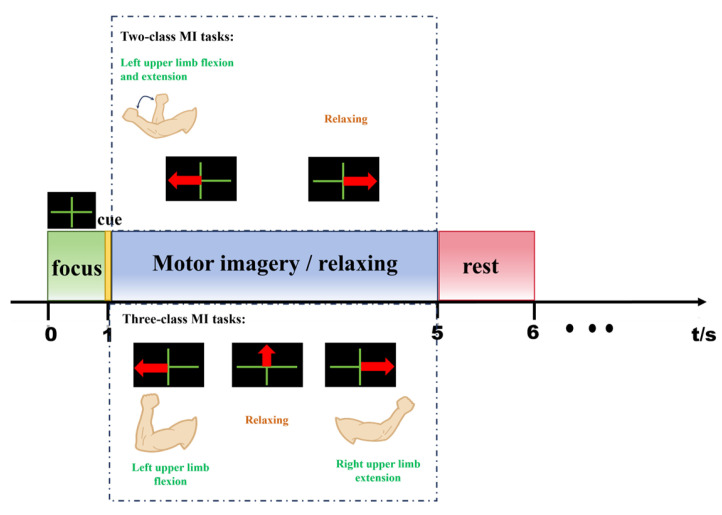
Experimental paradigm.

To evaluate the performance of the DFBCSP algorithm under different complexity levels, this study designed two-class and three-class MI experiments. The two-class task included left upper limb flexion and extension and relaxing state, with 10 blocks and a total of 200 trials; the three-class task comprised left upper limb flexion, right upper limb extension, and relaxing state, involving 10 blocks and a total of 300 trials. The task design was intended to elicit distinct ERD/ERS features, thereby providing a high-quality neurophysiological foundation for multi-band decomposition and spatial feature extraction of the DFBCSP algorithm. The experimental timing sequence is illustrated in Figure 3: at t = 0 s, a green cross was displayed to guide subjects into a focused state and calibrate the baseline signal; at t = 1 s, a target task was prompted via icons, for the two-class task, a red left-pointing arrow corresponded to left upper limb flexion and extension, and a red right-pointing arrow to the relaxing state; for the three-class task, a red left-pointing arrow denoted left upper limb flexion, a red right-pointing arrow stood for right upper limb extension, and a red upward-pointing arrow represented the relaxing state; subjects performed a 4 s MI from t = 1 s to t = 5 s, and the electroencephalographic signals in this time window served as the core input for the DFBCSP algorithm. Random inter-task rest periods were set between consecutive tasks. To suppress the interference of physiological artifacts on filter training, subjects maintained complete bodily stillness throughout the experiment to eliminate electromyography (EMG) interference; additionally, they minimized blinking and swallowing within the task window to inhibit physiological artifacts such as electrooculography (EOG).

### 2.2. EEG Data Preprocessing

We first performed Independent Principal Component Analysis (IPCA) on the continuous EEG signals; artifactual components such as electrooculographic (EOG) and electromyographic (EMG) artifacts were identified and removed by analyzing the temporal waveforms, scalp topographies, and power spectra of each component, after which the remaining components were back-projected to reconstruct artifact-free EEG signals. Subsequently, 4-s EEG segments were extracted from each trial as the full motor imagery (MI) cycle, and aberrant trials with a peak-to-peak amplitude exceeding ± 100 μV were excluded. Finally, zero-phase band-pass filtering at 4–40 Hz was applied to the extracted epochs using an 8th-order Butterworth band-pass filter to suppress low-frequency drifts and high-frequency noise, while preserving the mu (8–12 Hz) and beta (12–30 Hz) rhythmic components that primarily reflect MI-related event-related desynchronization (ERD) and event-related synchronization (ERS). The processed 4-s EEG segments were used as the input for subsequent DFBCSP feature extraction and classification.

### 2.3. Method for Feature Extraction

#### 2.3.1. Common Spatial Pattern

The basic principle of the CSP algorithm is to utilize matrix diagonalization to find a set of optimal spatial filters for projection, maximize the variance difference between the two types of signals, and thus obtain feature vectors with high discriminability.

Assume that $X_{1}$ and $X_{2}\;$ are the spatiotemporal signal matrices of multi-channel evoked responses in two-class MI tasks, respectively, both with a dimension of N×T, where *N* is the number of EEG channels, and *T* is the number of samples collected from each channel. To calculate their covariance matrices, it is assumed that *N* < *T*. For the two types of EEG MI tasks, a mathematical model of composite sources is generally adopted to describe the EEG signal. $X_{1}$ and $X_{2}$ can be expressed separately as follows:

(1) $$\begin{array}{c} X_{1}=\left[C_{1}\;C_{M}\right]\left[\begin{array}{c} S_{1}\\ S_{M} \end{array}\right]\; \end{array}\;,\begin{array}{c} {\;X}_{2}=\left[C_{2}\;C_{M}\right]\left[\begin{array}{c} S_{2}\\ S_{M} \end{array}\right] \end{array}\;$$

In Equation (1), $S_{1\;}\;$ and $S_{2\;}$ represent the two types of tasks, respectively. $S_{M}$ denotes the common source signal shared by the two types of tasks. $C_{1}$ and $C_{2}$ are composed of the common spatial patterns associated with $S_{1}$ and $S_{2}$. Since each spatial pattern is a vector of dimension *N* × 1, this vector is used to represent the distribution weight of the signal induced by a single source signal across *N* leads. $C_{M}$ denotes the common spatial pattern corresponding to $S_{M}$. The normalized covariance matrices $R_{1}$ and $R_{2}$ of $X_{1}$ and $X_{2}$ are expressed as follows, respectively:

(2) $$\begin{array}{c} \begin{array}{c} R_{1}=\frac{X_{1}X_{1}^{T}}{\mathrm{trace}\left(X_{1}X_{1}^{T}\right)} \end{array} \end{array}$$

(3) $$\begin{array}{c} \begin{array}{c} R_{2}=\frac{X_{2}X_{2}^{T}}{\mathrm{trace}\left(X_{2}X_{2}^{T}\right)} \end{array} \end{array}$$

$X^{T}$ denotes the transpose of the matrix X, and trace(X) denotes the sum of the diagonal elements of the matrix X. Next, the mixed spatial covariance matrix R is calculated and subjected to eigenvalue decomposition:

(4) $$R=-{\overset{-}{R}}_{1}+-{\overset{-}{R}}_{2}\;,\;R=U\lambda U^{T}\;$$

${\overset{-}{R}}_{i}(i$ = 1,2) denotes the average covariance matrix for the experiments of Task 1 and Task 2, respectively. U is the eigenvector matrix of the matrix λ, and λ is the diagonal matrix formed by the corresponding eigenvalues. The eigenvalues are then sorted in descending order. Perform the following transformation and decomposition on $R_{2}$ and $R_{2}$:

(5) $$P\;=\;\sqrt{\lambda^{-1}}U^{T}\;,\;S_{1}\;=\;PR_{1}P^{T}\;={\;B}_{1}{{\lambda_{1}B}_{1}}^{T}\;,\;S_{2}\;=\;PR_{2}P^{T}\;={\;B}_{2}{{\lambda_{2}B}_{2}}^{T\;}\;$$

From the above, it can be concluded that:

(6) $$B_{1}\;={\;B}_{2}\;=\;V\;,\;\lambda_{1}\;+{\;\lambda }_{2}=\;I\;$$

Since the sum of the eigenvalues of the two types of matrices is always 1, the eigenvector corresponding to the maximum eigenvalue of $S_{1}$ yields the minimum eigenvalue of $S_{2}$, and vice versa. When the eigenvalues in $\lambda_{1}$ are sorted in descending order, the corresponding eigenvalues in $\lambda_{2}$ are sorted in ascending order. Based on this, it can be inferred that $\lambda_{1}$ and $\lambda_{2}$ take the following forms:

(7) $$\lambda_{1}\;=\;\mathrm{diag}(I_{1}\;\sigma_{M}\;0),\;\lambda_{2}\;=\;\mathrm{diag}(0\;\sigma_{M}\;I_{2})\;$$

For the test data $x_{i}$, the corresponding projection matrix W (serving as the spatial filter) and its eigenvector $f_{i}$ are given as follows. By comparing $f_{i}$ with $f_{L}$ and $f_{R}$, the category of the *i*-th motor imagery can be determined.

(8) $${W=B^{T}P\;,\;Z}_{i}=W^{*}x_{i}\;,\;f_{i}=\frac{\mathrm{VAR}(Z_{i})}{\mathrm{sum}\left(\mathrm{VAR}(Z_{i})\right)}\;$$

#### 2.3.2. Discriminative Filter Bank Common Spatial Pattern

Traditional CSP algorithms typically extract features across a relatively broad frequency band (e.g., 4–40 Hz). However, the most discriminative neural oscillations, specifically the μ and β rhythms often reside within highly specific and narrow sub-bands that exhibit significant inter-subject and intra-subject variability. Utilizing a wide frequency range tends to introduce noise from irrelevant spectral components, which may obscure task-related information. Moreover, CSP filters are prone to overfitting when the available training samples are limited, leading to suboptimal generalization on novel datasets. The DFBCSP addresses these limitations systematically by integrating a filter bank architecture with discriminative feature selection strategies.

**Figure 4:**
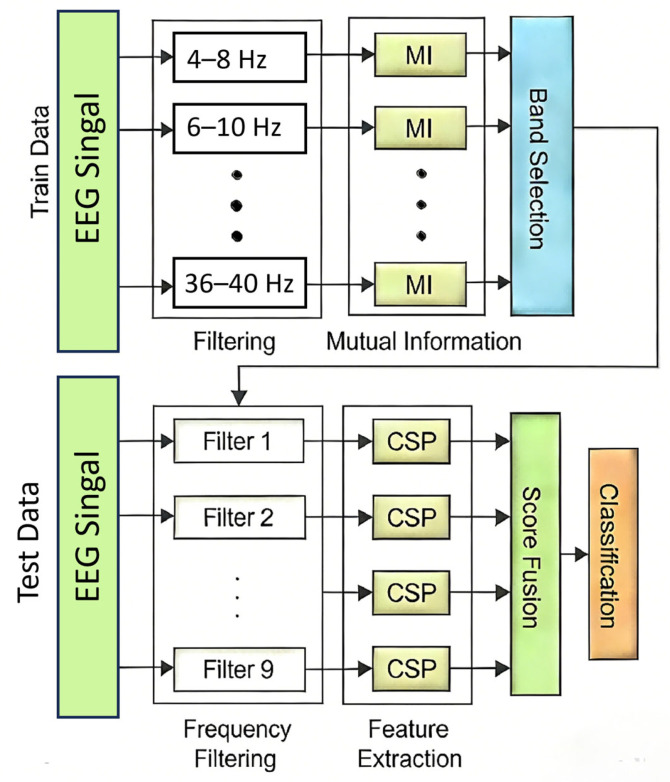
Flow chart of the DFBCSP algorithm processing.

In the study, the 4–40 Hz frequency band is decomposed into nine sub-bands using a bank of Butterworth filters, specifically: 4–8 Hz, 8–12 Hz, 12–16 Hz, 16–20 Hz, 20–24 Hz, 24–28 Hz, 28–32 Hz, 32–36 Hz, and 36–40 Hz. The methodology not only ensures the preservation of narrow-band components that may contain essential task-related information but also facilitates the extraction of band-specific spatial filter features. The flowchart of the DFBCSP algorithm is shown in Figure 4.

We employ the CSP algorithm to extract features from signal components that have been decomposed into multiple sub-bands. For the k(k=1,2…,9) frequency band, $x_{k,i}$ represents the test data in the *k*-th sub-frequency band during the *i*-th imagination. The proposed algorithm applies CSP independently to each $x_{k,i}$. Specifically, spatial covariance matrices are computed for the two task categories (Class 1 and Class 2) to determine the average normalized covariance matrices, denoted as $R_{k,1}$ and $R_{k,2}$, for each respective class within the k frequency band.

(9) $$\begin{array}{c} {\overset{-}{R}}_{k,1}=\mathrm{avg}{\left(\frac{X_{k,1}X_{k,1}^{T}}{\mathrm{trace}\left(X_{k,1}X_{k,1}^{T}\right)}\right)}_{Class1} \end{array}$$

(10) $$\begin{array}{c} {\overset{-}{R}}_{k,2}=\mathrm{avg}{\left(\frac{X_{k,2}X_{k,2}^{T}}{\mathrm{trace}\left(X_{k,2}X_{k,2}^{T}\right)}\right)}_{Class2} \end{array}$$

$X_{k,1}$ and $X_{k,2}$ represent the self-sub-band covariance matrices. The subsequent processing steps are similar to the CSP method described above:

(11) $$R_{k}={\overset{-}{R}}_{k,1}+{\overset{-}{R}}_{k,2},\;R_{k}=U_{k}\lambda_{k}U_{k}^{T}$$

(12) $$P_{k}=\sqrt{\lambda_{k}^{-1}}U_{k}^{T},\;S_{k,1}=P_{k}R_{k,1}P_{k}^{T},\;S_{k,2}=P_{k}R_{k,2}P_{k}^{T}$$

(13) $$W_{k}=B_{k}^{T}P_{k},\;Z_{k,i}=W_{k}x_{k,i}\;$$

(14) $$f_{k,i}=\frac{VAR(Z_{k,i})}{\sum VAR(Z_{k,i})}\;$$

In the final classification, we opted for mutual information rather than the Fisher Ratio method. Define $f_{k}=\left\{f_{1,i},\;f_{2,i},\ldots ,f_{9,i}\right\}$ as the feature set for sub-band k and $Y=\left\{y_{1},\;y_{2}\right\}$ as the label set. Discretize the continuous $f_{k,i}$ into n equal-width intervals, the mutual information is then computed as:

(15) $$MI_{k}=\sum_{f\in f_{k}}\sum_{y\in Y}p(f,y)\log_{2}\frac{p(f,y)}{p(f)p(y)}\;$$

Arrange the sub-bands by $MI_{k}$ in descending order, select the top m sub-bands with the highest mutual information, and concatenate them to produce the final feature vector:

(16) $$F_{k}={\left[f_{1}^{T},\;f_{2}^{T},\ldots ,\;f_{m}^{T}\right]}^{T\;}$$

Input the above $F_{k}$ into a classifier (such as SVM) to obtain discriminant scores. Finally, perform score fusion on the discriminant scores of the nine channels to get the final imagination classification.

### 2.4. Classification

To evaluate the efficacy of the feature extraction algorithms and construct robust EEG recognition models, the study employs three highly representative and widely utilized classifiers within the field of EEG signal processing: Multi-layer perceptron (MLP) [27], support vector machine (SVM) [28], and linear discriminant analysis (LDA) [29]. By coupling these classifiers with both DFBCSP and conventional CSP, we aim to comprehensively investigate the performance of distinct model configurations across two-class and three-class classification tasks.

#### 2.4.1. SVM

The primary objective of the SVM is to map input vectors into a high-dimensional feature space, constructing an optimal separating hyperplane within this space to achieve the precise partitioning of feature vectors. During the classification evaluation for the motor imagery tasks in this study, we compared the performance of linear, nonlinear, and polynomial kernel functions. Empirical results indicated that the linear kernel yielded the optimal performance; consequently, it was selected as the final kernel, with the penalty parameter C in the C-SVC classifier set to 1. For the feature $F_{k}\;\in {\;R}^{D\;}$ and the label y ∈ {−1,1}, learn the hyperplane $w^{T}F_{k}\;+\;b\;=\;0$. The optimization objective is as follows:

(17) $$\underset{w,b}{\min}\frac{1}{2}\|w{\|}^{2}\;s.t.\;y_{i}(w^{⊤}F_{k,i}+b)\geq 1\;(\forall i)\;$$

Then introduce the Lagrange multipliers $\alpha_{i}\;\geq \;0$ and transform it into a dual problem:

(18) $$\underset{\alpha }{\max}\sum_{i\;=\;1}^{N}\alpha_{i}-\frac{1}{2}\sum_{i,j\;=\;1}^{N}\alpha_{i}\alpha_{j}y_{i}y_{j}K(F_{k,i},F_{k,j})\;s.t.\;\sum_{i=1}^{N}\alpha_{i}y_{i}=0$$

$K(F_{k,i},F_{k,j})$ is kernel function. After solving, the classifier is obtained:

(19) $$f_{SVM}(F_{k})=sign\left(\sum_{i\;=\;1}^{N}\alpha_{i}y_{i}K(F_{k,i},F_{k})+b\right)\;$$

The bias b is determined by the support vector samples. Fuse (e.g., average) the SVM discriminant scores of the 9 channels and output the final classification.

#### 2.4.2. MLP

MLP represents a quintessential feedforward artificial neural network characterized by robust nonlinear mapping capabilities, enabling the capture of intricate functional relationships inherent in EEG signal features. In this study, the MLP classifier is structured with an input layer, a hidden layer, and an output layer. We configured a hidden layer comprising 64 neurons and adopted the rectified linear unit as the activation function to expedite gradient convergence. The model parameters are updated via the Backpropagation algorithm combined with the Adam optimizer, utilizing the cross-entropy loss function to minimize classification error. The specific process is as follows. Here, f*,* h, and $\hat{y}$ are the outputs of the input layer, hidden layer, and output layer, respectively. $W_{1}$ and $W_{2}$ are the corresponding parameter matrices. $b_{1}$ and $b_{2}$ are the corresponding biases, and L is the cross–entropy loss.

(20) $$h=\;\max\left(0,\;f\right)=\max\left(0,\;W_{1}F_{k}+b_{1}\right)\;$$

(21) $$\hat{y}=\frac{1}{1\;+\exp\left(-\left(W_{2}h+b_{2}\right)\right)}\;$$

(22) $$L=-\frac{1}{N}\sum_{i=1}^{N}\left[y_{i}{\log\hat{y}}_{i}+(1-y_{i})\log\left(1-{\hat{y}}_{i}\right)\right]\;$$

#### 2.4.3. LDA

LDA’s fundamental principle involves identifying the optimal projection direction to reduce the dimensionality of high-dimensional data, ensuring that the transformed low-dimensional representation satisfies the discriminant criterion of minimizing intra-class scatter while maximizing inter-class scatter. In this study, we employ the classical LDA algorithm configured with an automatic shrinkage estimator to enhance the model’s generalization capability in scenarios involving limited sample sizes. The specific process is as follows. Here, $\mu_{c}$, $S_{w}$, $S_{b}$, w, and $\hat{y}$ represent the class mean, within—class scatter, between—class scatter, optimal projection direction, and final decision output, respectively.

(23) $$\mu_{c}=\frac{1}{N_{c}}\sum_{i=1}^{N_{c}}F_{k,i}^{\left(c\right)}\;$$

(24) $$S_{w}=\sum_{c=1}^{2}\sum_{i=1}^{N_{c}}\left(F_{k,i}^{\left(c\right)}\;-\;\mu_{c}\right){\left(F_{k,i}^{\left(c\right)}\;-\;\mu_{c}\right)}^{T\;}\;$$

(25) $$S_{b}=\left(\mu_{1}\;-{\;\mu }_{2}\right){\left(\mu_{1}\;-{\;\mu }_{2}\right)}^{T}\;$$

(26) $$w=S_{w}^{-1}\left(\mu_{1}\;-\;\mu_{2}\right)\;$$

(27) $$\hat{y}=\mathrm{sign}\left(w^{T}F_{k}\;-\;\frac{w^{T}\left(\mu_{1}+\mu_{2}\right)}{2}\right)\;$$

## 3. Results and Analysis

**Figure 5:**
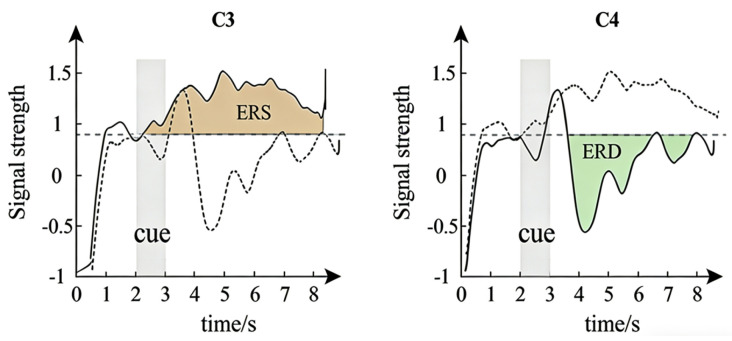
The time course of ERD/ERS from second 0 to 8.

Figure 5 first shows the change process of ERD/ERS from 0 s to 8 s. There are obvious ERD and ERS in the electroencephalogram cortical signals, and the time range for the occurrence of ERD/ERS in each experiment is from 3.5 s to 5.5 s. When imagining the flexion and extension movements of the left upper limb, the C3 channel shows a higher potential during the ERS phenomenon, while the C4 channel shows a lower potential during the ERD phenomenon, which reflects the dynamic neural regulation of the brain when processing information.

To facilitate optimal experimental outcomes, the dataset is evenly divided into five subsets. Each time, four subsets are used as the training set and one subset is used as the validation set to conduct a five—fold cross—validation experiment. The specific training accuracies for the respective classification tasks are detailed in Table 1 and Table 2. Subsequently, the performance of six algorithm combinations—comprising DFBCSP + MLP, DFBCSP + SVM, DFBCSP + LDA, CSP + MLP, CSP + SVM, and CSP + LDA—was rigorously evaluated on the test set using classification accuracy, the Kappa coefficient, and receiver operating characteristic (ROC) curves.

For subjects S1–S16, we use the average accuracy and standard deviation in the table to calculate the 95% confidence interval for each algorithm. The calculation formula is as follows:

(28) $$\mathrm{confidence}\;\mathrm{interval}\;=\;\overset{-}{x}\pm t_{\frac{\alpha }{2},\;n-1}\cdot \frac{s}{\sqrt{n}}\;$$

Here, $\overset{-}{x}$ represents the accuracy rate, n is the number of samples, s is the standard deviation, and the degrees of freedom $t_{\frac{\alpha }{2},\;n-1}$.

To quantify the differences in classification performance among the various algorithms, we first conducted a one-way repeated-measures analysis of variance (ANOVA [30]) on the classification accuracies of 16 subjects obtained with the six algorithms. The results demonstrated an extremely significant main effect of algorithm type on classification accuracy, indicating the presence of overall differences in the accuracies of the six algorithms, as detailed in Table 3 and Table 4. To further identify the specific sources of these differences, we performed post hoc multiple comparisons using Bonferroni-corrected paired *t*-tests. The test results showed that the DFBCSP series algorithms achieved significantly higher accuracies than their corresponding conventional CSP series algorithms. Additionally, under the DFBCSP framework, the Multi-Layer Perceptron (MLP) classifier significantly outperformed the Support Vector Machine (SVM) and Linear Discriminant Analysis (LDA) classifiers, which confirms the performance superiority of the DFBCSP + MLP algorithm.

To conduct an in-depth analysis of the classification performance of each algorithm, we introduced the Kappa coefficient as a pivotal evaluation metric. An elevation in this value directly reflects enhancements in both model accuracy and stability [31]. Drawing upon experimental data from subjects S1 through S16, Table 5 and Table 6 delineate the specific performance of the six comparative methods across two-class and three-class classification tasks, respectively. Through the comparison of these quantitative data, performance disparities among the models when addressing classification tasks of varying complexities can be clearly observed.

**Figure 6:**
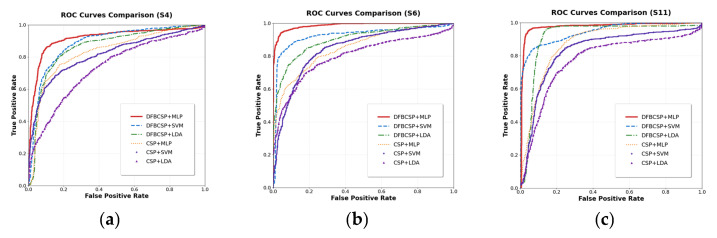
ROC curves of the six classification methods in the two-class classification task: (**a**) results for subject S4; (**b**) results for subject S6; (**c**) results for subject S11.

**Figure 7:**
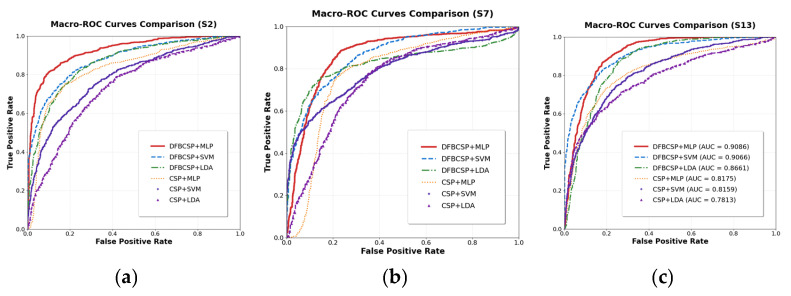
Macro-ROC curves of the six classification methods in the three-class classification task: (**a**) results for subject S2; (**b**) results for subject S7; (**c**) results for subject S13.

The ROC curve not only dynamically depicts the trade-off between the true positive rate (TPR) and false positive rate (FPR) across a continuum of decision thresholds but also offers a robust characterization of a model’s discriminative performance in motor imagery electroencephalography (MI-EEG) classification. In this study, clear classification labels were defined a priori: for the two-class task, MI of left upper limb extension/flexion was designated as the positive class, while the resting (relaxation) state was assigned as the negative class. For the three-class task, to enable a streamlined evaluation of multidimensional discriminative performance and objectively reflect the model’s overall ability to recognize the three specific movement states—Relaxation, Left Limb Flexion, and Right Limb Extension—we employed the macro-average ROC curve, which is derived by macro-averaging the per-class ROC curves computed for each individual class against all other classes combined. As a canonical global metric for assessing classifier performance, the AUC is highly sensitive to the distinctness of the learned classification boundaries; an AUC value approaching 1 (corresponding to an ROC curve that nears the top-left corner of the coordinate plane) signifies the model’s superior capacity to extract and discriminate task-relevant MI-EEG signal features [32]. Figure 6 and Figure 7 present the ROC curve profiles for representative subjects across different experimental paradigms, while Table 7 reports the mean AUC values (including the macro-average AUC for the three-class task) for each classification method.

Based on the data in the above pictures and tables, it can be concluded that the DFBCSP + MLP algorithm demonstrates significant performance advantages when processing EEG signals. In summary, the DFBCSP + MLP algorithm demonstrated superior classification performance in the task of upper-limb-motor-intention recognition; comparative experiments indicate that this algorithm achieved a significant improvement in performance relative to the conventional CSP algorithm.

**Figure 8:**
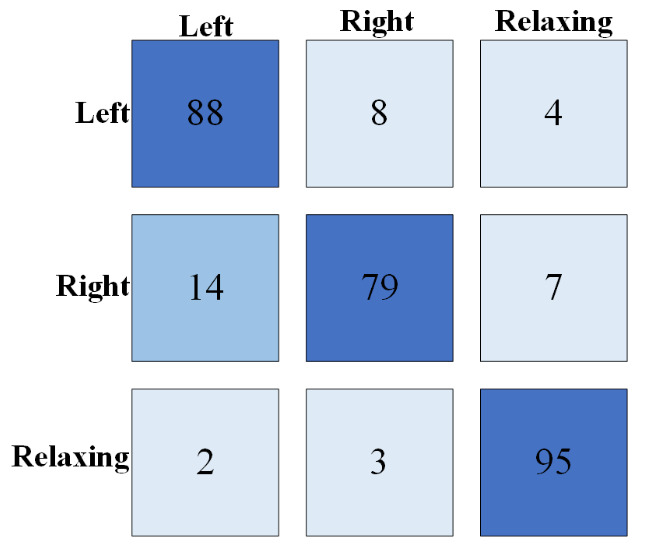
Three-class classification confusion matrix.

Figure 8 shows the confusion matrix of the real-time three-classification results for one of the subjects. The main diagonal of the confusion matrix reflects the prediction of the three categories. The columns represent the actual categories, and the rows represent the predicted categories. The darker the color, the higher the prediction accuracy.

## 4. Discussion

The performance of DFBCSP and conventional CSP in motor imagery (MI) EEG decoding is systematically evaluated through two-class (relaxing vs. left upper limb MI) and three-class tasks, with results corroborated by accuracy, Kappa coefficient, ROC curves, and AUC metrics.

Experimental results demonstrate that the selection of feature extraction methods and classifiers exerts a significant influence on the recognition performance of electroencephalogram (EEG) signals. In terms of feature extraction, DFBCSP exhibited superior performance to the traditional CSP in both binary and ternary classification tasks, with an average accuracy improvement of approximately 5% to 8% and a significantly lower standard deviation. This verifies the robustness of multi-band discriminative features in capturing the non-stationary information of EEG signals and suppressing individual differences. In the aspect of classifiers, under the same feature conditions, the performance of the three classifiers followed the order of MLP > SVM > LDA. This reflects that nonlinear classifiers represented by MLP possess stronger feature mapping and discriminative capabilities than linear classifiers (LDA) in processing high-dimensional EEG features. In summary, the DFBCSP + MLP combination achieved the highest recognition accuracy in both binary (94.83%) and ternary (86.20%) classification tasks, with the most concentrated distribution of 95% confidence intervals. This proves that the combination serves as the optimal recognition framework for addressing complex multi-class EEG tasks.

BCI illiteracy refers to the phenomenon in motor imagery (MI) based brain–computer interface (BCI) training and experiments where certain individuals fail to achieve sufficiently high and usable control performance over an extended period [33]. Its causes may be associated with multiple factors, such as neurophysiological differences, yet no unified conclusion has been reached to date. In the experiments, Subject S4 exhibited a significantly lower accuracy rate than other participants in the ternary classification task, presenting this phenomenon in a typical manner. The DFBCSP method can amplify weak and scattered discriminative information through multi-band filtering and mutual information-based discriminative feature selection. Even for Subject S4, the DFBCSP combined with a multi-layer perceptron (MLP) achieved an accuracy increase of approximately 10.38% compared with the CSP + MLP framework in the three-class task; in two-class and three-class tasks, it further yielded increases of 16.92% and 15.54%, respectively, relative to the CSP + LDA framework. This demonstrates the DFBCSP’s robustness and compensatory ability for users with weak signals/low controllability, and its potential to enhance the generalizability of BCI systems to illiterate subjects. This study only investigates the manifestations and performance differences of BCI illiteracy, without exploring its underlying neural mechanisms or enabling a rigorous clinical diagnosis of BCI illiteracy. Moreover, DFBCSP cannot fundamentally eradicate this phenomenon. Future research will conduct in-depth investigations into the causes and intervention strategies of BCI illiteracy, analyze the characteristics of low-controllability users via multi-dimensional assessment, and explore the improvement effects of combined methodological frameworks.

Regarding the computational complexity and system response time, we have specifically presented them in Table 8. Here, *N*, *K*, *T*, *F*, *S*, and *H*, respectively, represent the number of EEG signal channels, the number of sub-bands of DFBCSP, the number of sampling points of the EEG signal, the number of extracted features, the number of training samples, and the number of neurons in the hidden layer of the MLP.

While the proposed approach yields promising results, several limitations merit discussion. First, the study’s sample size (sixteen participants) is relatively small, and the MI tasks (e.g., left flexion vs. right extension) are simplified compared to real-world rehabilitation scenarios. Future research should expand the cohort to include more diverse populations and validate the method on more complex, ecologically valid MI paradigms. Second, the current DFBCSP framework uses a fixed set of nine sub-bands, which may not be optimal for all individuals or MI tasks. Adaptive sub-band selection (e.g., personalized frequency partitioning based on individual EEG characteristics) could further enhance performance, particularly for subjects with “BCI illiteracy”.

In terms of clinical relevance and translational potential, the method’s feasibility hinges on addressing key real-world constraints. Temporally, the DFBCSP + MLP’s offline processing latency is acceptable for offline personalized rehabilitation planning but requires optimization (e.g., adaptive sub-band pruning, lightweight MLP quantization) to meet the <100 ms latency threshold for real-time BCI-guided training, critical for patient-machine interaction in clinical settings. System-wise, the moderate computational demands (Table 8) are compatible with portable EEG devices, supporting deployment in rehabilitation centers or home-based care, though power consumption optimization is needed for long-term wearable use. Clinically, the simplified MI tasks limit direct translation; future validation on patients with upper limb dysfunction should incorporate functional, task-specific movements (e.g., reaching, grasping) to align with real rehabilitation goals.

In recent years, deep learning methods like CNNs and their variants [34,35] have achieved excellent performance on public MI-EEG datasets by automatically learning spatiotemporal features from raw or minimally preprocessed signals in an end-to-end manner, showing great potential for modeling complex nonlinear EEG patterns yet relying on sufficient data and computing power; by contrast, the DFBCSP + MLP proposed in this study integrates classic neurophysiological priors with a lightweight nonlinear classifier, where DFBCSP leverages μ and β band ERD/ERS priors and improves feature interpretability and robustness via multi-sub-band filtering and discriminative feature selection, and MLP implements nonlinear mapping at the feature space level with far fewer parameters than deep CNNs/EEGNet. This method has lower demands for training data scale and hardware resources, making it more suitable for the few-shot learning, portability and real-time performance requirements of rehabilitation scenarios; for future work, we will further incorporate lightweight CNNs/EEGNet or attention modules into the framework to build a “DFBCSP features + micro deep networks” hybrid architecture, and integrate transfer learning and adaptive frequency band selection strategies to compare and fuse the performance and deployability of mainstream deep learning methods in clinical populations. Notably, the present study does not incorporate the integration of physical modeling (e.g., the finite element method, FEM) with artificial intelligence (AI) into its research scope, yet numerous studies have demonstrated that this integration strategy can effectively enhance the generalization ability and interpretability of AI systems for upper limb rehabilitation [36]. This presents a highly promising future research direction for further improving the interpretability and robustness of rehabilitation-based brain–computer interface (BCI) systems based on the method we proposed.

Despite these limitations, the findings highlight DFBCSP’s potential as a robust feature extraction tool for upper limb MI-EEG decoding, providing a technical foundation for precision neurorehabilitation. For clinical applications, the DFBCSP + MLP model could be integrated into wearable BCI devices to deliver personalized rehabilitation training, particularly for patients with upper limb motor dysfunction.

## 5. Conclusions

We investigated six algorithm combinations to optimize the classification strategy and performance of the MI-BCI system by differentiating between upper limb extension and flexion movements. These combinations included DFBCSP + MLP, DFBCSP + SVM, and DFBCSP + LDA, alongside the conventional CSP + based counterparts: CSP + MLP, CSP + SVM, and CSP + LDA. By incorporating multi-band filtering and mutual information-based discriminative feature selection, the DFBCSP algorithm effectively eliminates redundant information while retaining features that yield the highest classification contribution. Methodologies employing the DFBCSP algorithm consistently outperformed those based on the conventional CSP algorithm. Specifically, in the two-class classification task, the DFBCSP + MLP method achieved a remarkable average accuracy of 94.83%, representing an improvement of approximately 8.23% over traditional methods; the average Kappa coefficient reached 0.890, and the average AUC value was 0.954. Furthermore, in the three-class classification task, the Kappa coefficient improved by approximately 0.10. Moreover, our results indicate that the MLP classifier, leveraging its nonlinear mapping capabilities, exhibited performance significantly superior to that of SVM and LDA. Consequently, identified as the optimal combination of spatial filtering and classification algorithms, the DFBCSP + MLP method demonstrates immense potential for enhancing the performance of upper limb motor imagery systems.

## Figures and Tables

**Table 1:** Accuracy (%) of different methods (test set) in a two-class classification task.

Subject	Method					
	CSP + LDA	CSP + SVM	CSP + MLP	DFBCSP + LDA	DFBCSP + SVM	DFBCSP + MLP
S1	92.57	93.64	92.78	94.27	96.36	98.75
S2	87.62	92.33	93.46	91.75	92.05	95.28
S3	83.14	84.51	86.3	85.27	91.94	95.48
S4	74.95	79.25	81.37	84.62	87.45	91.87
S5	78.38	80.34	83.61	86.45	89.83	93.24
S6	81.82	82.74	83.54	86.21	90.59	93.66
S7	84.57	85.49	84.92	87.73	89.47	94.08
S8	86.48	88.73	91.23	91.39	93.76	97.34
S9	79.56	81.46	81.77	84.69	87.51	92.57
S10	85.79	87.34	88.42	90.94	91.76	94.84
S11	84.63	86.85	85.68	88.64	92.45	95.18
S12	80.62	83.05	85.41	84.75	87.68	93.71
S13	82.37	83.77	84.37	85.76	90.16	94.14
S14	79.74	81.19	82.64	84.98	88.78	93.71
S15	85.31	88.54	89.79	90.29	93.46	96.59
S16	89.48	90.62	91.34	92.44	92.97	96.87
Average accuracy (%)	83.56 ± 4.30	85.62 ± 4.20	86.60 ± 3.85	88.14 ± 3.15	91.01 ± 2.45	94.83 ± 1.79
95% confidence interval (%)	(81.29, 85.83)	(83.43, 87.81)	(84.58, 88.62)	(86.38, 89.90)	(89.68, 92.34)	(93.78, 95.88)

**Table 2:** Accuracy (%) of different methods (test set) in three-class classification tasks.

Subject	Method					
	CSP + LDA	CSP + SVM	CSP + MLP	DFBCSP + LDA	DFBCSP + SVM	DFBCSP + MLP
S1	76.41	76.85	79.37	82.51	82.94	85.73
S2	76.62	78.39	81.54	80.23	81.76	86.41
S3	78.83	81.49	83.71	82.74	84.61	88.25
S4	63.59	65.64	70.28	71.35	74.24	79.13
S5	77.42	76.83	79.41	81.54	83.62	84.39
S6	68.51	71.45	74.84	75.69	80.37	83.46
S7	67.27	70.6	73.16	74.37	77.82	82.37
S8	73.39	76.31	79.52	81.25	85.39	90.29
S9	80.31	79.87	81.33	79.91	80.17	85.57
S10	69.84	74.62	79.25	80.34	83.51	87.64
S11	78.25	81.13	83.61	87.58	89.77	92.74
S12	67.43	69.57	74.25	78.36	83.04	85.36
S13	79.22	78.46	80.34	79.77	85.27	87.18
S14	68.31	70.35	75.43	79.85	80.79	84.74
S15	66.58	69.71	71.76	76.97	79.64	83.53
S16	81.47	82.79	84.39	87.79	91.57	92.48
Average accuracy (%)	73.34 ± 5.67	75.25 ± 4.96	78.26 ± 4.28	80.02 ± 4.13	82.78 ± 4.10	86.20 ± 3.47
95% confidence interval (%)	(70.28, 76.40)	(72.72, 77.78)	(76.02, 80.50)	(77.85, 82.19)	(79.94, 84.62)	(84.25, 88.15)

**Table 3:** Significance testing for two-class classification results.

Value *p*, Value t (df = 15)	CSP + LDA	CSP + SVM	CSP + MLP	DFBCSP + LDA	DFBCSP + SVM	DFBCSP + MLP
CSP + LDA	/	t = 1.73, *p* = 0.295	t = 3.15, *p* < 0.05	t = 6.34, *p* < 0.05	t = 9.27, *p* < 0.05	t = 12.15, *p* < 0.05
CSP + SVM	/	/	t = 1.42, *p* = 0.407	t = 5.58, *p* < 0.05	t = 8.51, *p* < 0.05	t = 11.38, *p* < 0.05
CSP + MLP	/	/	/	t = 4.82, *p* < 0.05	t = 7.75, *p* < 0.05	t = 10.62, *p* < 0.05
DFBCSP + LDA	/	/	/	/	t = 5.63, *p* < 0.05	t = 7.45, *p* < 0.05
DFBCSP + SVM	/	/	/	/	/	t = 4.89, *p* < 0.05

**Table 4:** Significance testing for three-class classification results.

Value *p*, Value t (df = 15)	CSP + LDA	CSP + SVM	CSP + MLP	DFBCSP + LDA	DFBCSP + SVM	DFBCSP + MLP
CSP + LDA	/	t = 1.59, *p* = 0.367	t = 2.94, *p* < 0.05	t = 5.82, *p* < 0.05	t = 8.45, *p* < 0.05	t = 10.87, *p* < 0.05
CSP + SVM	/	/	t = 1.35, *p* = 0.438	t = 5.07, *p* < 0.05	t = 7.71, *p* < 0.05	t = 10.12 *p* < 0.05
CSP + MLP	/	/	/	t = 4.31, *p* < 0.05	t = 6.96, *p* < 0.05	t = 9.37, *p* < 0.05
DFBCSP + LDA	/	/	/	/	t = 5.03, *p* < 0.05	t = 6.74, *p* < 0.05
DFBCSP + SVM	/	/	/	/	/	t = 4.26, *p* < 0.05

**Table 5:** Kappa coefficients of a two-class classification method.

Subject	Method					
	CSP + LDA	CSP + SVM	CSP + MLP	DFBCSP + LDA	DFBCSP + SVM	DFBCSP + MLP
S1	0.734	0.749	0.777	0.798	0.826	0.861
S2	0.652	0.687	0.712	0.762	0.857	0.906
S3	0.738	0.767	0.786	0.817	0.834	0.875
S4	0.631	0.685	0.725	0.785	0.821	0.881
S5	0.702	0.714	0.742	0.783	0.832	0.893
S6	0.655	0.697	0.721	0.794	0.836	0.884
S7	0.724	0.741	0.793	0.824	0.857	0.912
S8	0.708	0.722	0.761	0.811	0.852	0.896
S9	0.687	0.713	0.748	0.787	0.838	0.892
S10	0.741	0.789	0.814	0.861	0.892	0.921
S11	0.722	0.753	0.792	0.824	0.871	0.931
S12	0.652	0.686	0.727	0.765	0.813	0.856
S13	0.665	0.714	0.734	0.767	0.807	0.867
S14	0.737	0.751	0.781	0.812	0.846	0.878
S15	0.648	0.687	0.716	0.758	0.818	0.863
S16	0.711	0.769	0.81	0.835	0.875	0.924
Average Kappa coefficient	0.694 ± 0.04	0.727 ± 0.03	0.759 ± 0.03	0.799 ± 0.03	0.842 ± 0.02	0.890 ± 0.02

**Table 6:** Kappa coefficients of three-class classification methods.

Subject	Method					
	CSP + LDA	CSP + SVM	CSP + MLP	DFBCSP + LDA	DFBCSP + SVM	DFBCSP + MLP
S1	0.647	0.659	0.69	0.719	0.726	0.772
S2	0.594	0.627	0.658	0.703	0.739	0.793
S3	0.615	0.643	0.674	0.712	0.725	0.759
S4	0.462	0.524	0.602	0.637	0.693	0.757
S5	0.544	0.603	0.614	0.633	0.688	0.762
S6	0.623	0.648	0.696	0.722	0.747	0.806
S7	0.493	0.544	0.618	0.627	0.651	0.734
S8	0.484	0.527	0.609	0.635	0.662	0.748
S9	0.513	0.572	0.624	0.674	0.713	0.771
S10	0.537	0.571	0.618	0.662	0.71	0.763
S11	0.521	0.593	0.629	0.668	0.734	0.788
S12	0.473	0.542	0.617	0.649	0.681	0.734
S13	0.583	0.615	0.654	0.714	0.762	0.815
S14	0.647	0.673	0.708	0.728	0.753	0.803
S15	0.524	0.568	0.628	0.691	0.737	0.807
S16	0.497	0.557	0.61	0.677	0.721	0.784
Average Kappa coefficient	0.546 ± 0.06	0.592 ± 0.04	0.641 ± 0.03	0.678 ± 0.03	0.715 ± 0.03	0.775 ± 0.02

**Table 7:** Average AUC values of different classification methods.

AUC Value	Method					
	CSP + LDA	CSP + SVM	CSP + MLP	DFBCSP + LDA	DFBCSP + SVM	DFBCSP + MLP
Two-class classification (average)	0.795	0.833	0.857	0.894	0.928	0.954
Three-class classification (macro-average)	0.754	0.781	0.803	0.847	0.862	0.897

**Table 8:** Analysis of computational complexity and response time.

	Method					
	CSP + LDA	CSP + SVM	CSP + MLP	DFBCSP + LDA	DFBCSP + SVM	DFBCSP + MLP
Computational Complexity	*O* (*N*^3^ + *T* × *N*^2^ + *F*^3^)	*O* (*N*^3^ + *T* × *N*^2^ + *S*^2^ × *F*)	*O* (*N*^3^ + *T* × *N*^2^ + *S* × *F* × *H*)	*O* (*K* × (*N*^3^ + *T* × *N*^2^) + *F*^3^)	*O* (*K* × (*N*^3^ + *T* × *N*^2^) + *S*^2^ × *F*)	*O* (*K* × (*N*^3^ + *T* × *N*^2^) + *S* × *F* × *H*)
Response time	0.3–0.5 s	0.3–0.5 s	0.3–0.5 s	0.3–0.5 s	0.3–0.5 s	0.3–0.5 s

## Data Availability

Data is available from the lead contact upon request.

## Abbreviations

The following abbreviations are used in this manuscript:

BCI	Bain–Computer Interfaces
CSP	common spatial pattern
DFBCSP	Discriminative Filter Bank Common Spatial Pattern
MI	motor imagery
MLP	Multilayer Perceptron
SVM	Support Vector Machine
LDA	Linear Discriminant Analysis
ALS	Amyotrophic Lateral Sclerosis
SCI	Spinal Cord Injury
ERD	Event Related Desynchronization
ERS	asevent-related synchronization
EMG	Electromyogram
EOG	Electrooculogram
ROC	Receiver Operating Characteristic
TPR	True Positive Rate
FPR	False Positive Rate
AUC	Area Under the Curve
